# Dignity of patients with palliative needs in the Middle East: an integrative review

**DOI:** 10.1186/s12904-021-00791-6

**Published:** 2021-07-16

**Authors:** Silva Dakessian Sailian, Yakubu Salifu, Rima Saad, Nancy Preston

**Affiliations:** 1 American University of Beirut, Hariri School of Nursing, Riad El Solh, PO Box: 11 0236, Beirut, 1107 2020 Lebanon; 2 International Observatory on End of Life Care, Division of Health Research, Faculty of Health and Medicine, Sir John Fisher Drive, Lancaster University, Lancaster, LA1 4YW UK

**Keywords:** Dignity, Palliative care, Integrative review, Middle East, Chochinov model

## Abstract

**Background:**

Patients with palliative needs experience high psychological and symptom distress that may lead to hopelessness and impaired sense of dignity. Maintaining patient dignity or the quality of being valued is a core aim in palliative care. The notion of dignity is often explained by functionality, symptom relief and autonomy in decision making. However, this understanding and its implications in Middle Eastern countries is not clear.

The aim of this review is to 1) explore the understanding of dignity and how dignity is preserved in adult patients with palliative care needs in the Middle East 2) critically assess the findings against the Dignity Model dominant in western literature.

**Method:**

Using an integrative review we searched four databases EMBASE, Psych-Info, CINAHL, and PubMed. These databases retrieve a broad literature on palliative care and are often chosen in other palliative care reviews. To enhance the search strategy, three online journals were hand searched, reference lists of review papers scanned, and forward citations sought. No time limits were applied. The retrieved papers were assessed independently by two authors including quality assessment using the Hawker’s appraisal tool.

**Results:**

Out of the 5113 studies retrieved, 294 full texts were assessed. Sixteen studies were included for synthesis of which fourteen were published in Iran. Seven themes were developed after data analysis: Maintaining Privacy and Secrecy; Gentle communication with a dialogue that preserves hope instead of blunt truth-telling; Abundance characterised by accessibility to medical supplies and financial stability; Family Support where relatives deliver major assistance in care; Physical Fitness; Reliable health care, and Social justice that endorses equal care to all.

**Conclusion:**

The results are compatible with the existing evidence from the Dignity Model ascertaining that dignity is socially mediated and influenced by interactions and physical fitness. Nevertheless, the findings highlight that patient dignity is also shaped by the socio-political, cultural, and economic conditions of the country, where family support, gentle communication and accessible health care are essential elements.

## Introduction

Patients with advanced chronic or terminal illnesses experience high psychological and symptom distress that may lead to hopelessness and sometimes an impaired sense of dignity [1]. Maintaining the dignity of patients who are stressed by the various demands and changes that chronic and advanced illnesses impose on their daily lives is a core aspect of palliative care philosophy [2, 3]. The purpose of this systematic review was to explore and synthesise the research evidence on the understanding of dignity of patients with chronic and life-threatening illnesses in the Middle Eastern context. An empirical model of dignity developed by Chochinov, constructed by interviewing end stage cancer patients in Canada, [4] described dignity at end of life in three broad categories of ‘illness-related concerns’, ‘dignity conserving repertoire’; and ‘social dignity inventory’. This model has been instrumental in developing dignity preserving interventions for patients and families [5]. Parallel to the Chochinov findings, dignity of patients with palliative care needs has been related to functionality, symptom relief and autonomy over the dying process [6, 7]. However, this understanding and its implications in Middle Eastern countries that is characterised a religious and family-oriented culture and often affected by political turmoil, is not clear.

Palliative care resources are scarce in Arab-Muslim populated countries in the Middle East [8]. The availability and access to opioids to alleviate the pain of patients with terminal or advanced illnesses is restricted through local legislation [9]. The integration of palliative care in the national health plan and its acknowledgment as a separate discipline is limited rendering palliative care services fragmented and funding scarce [10]. Palliative care education is not fully incorporated into medical and nursing educational curricula leading to a shortage of well-trained palliative care specialists [9–11], and consequently limiting the access to quality palliative care. The absence of national laws regulating advance decision such as “do not resuscitate” orders and the inconsistent definition of brain death in Muslim countries presents a hindrance to the development and provision of palliative care, advanced care planning and consequently protecting patient dignity [11]. Often the unstable geopolitical, economic, and the mosaic sociocultural fabric of the region that is under a strong influence of religion, traditions, and culture, shapes the understanding of human dignity in general and subsequently in palliative care settings which then affects policy development and dignity of patients [12, 13].

Since family role and religion are recurrent values that envelope the middle eastern individual and has overarching implications in preserving patient dignity [14], it is intriguing to delve into the embedded facets of patient dignity to identify its nuances in the Middle East region in comparison to other regions and cultures and particularly to the dominant Western understanding. Upholding patient dignity is a core ethical value in nursing [15] as well as medical practice [16]. Thus, an explicit clarity of this sensitive phenomenon would inform health care providers and educators to attend to and protect the dignity related needs of palliative care patients with cultural humility. No published systematic review has been found in the Middle Eastern context that addresses the concept of dignity in patients with palliative care needs. Therefore, this review is unique, it unfolds pertinent knowledge for all health stakeholders, and paves the way for better clinical practice and future research.

### The review question and aim

What is the understanding of dignity and how is dignity preserved in patients with palliative care needs in the Middle East?

The overarching aim of this review is to explore and critically synthesize the evidence on the concept of personal dignity in the Middle Eastern palliative care setting from the perspective of the patient, health care provider, and family caregiver, examining how dignity is enhanced or undermined while receiving care. This review looks at:

1. The perceptions of the concept of human dignity from the perspective of the patient, family caregiver, or health care provider within adult palliative care in the Middle Eastern region.
2. Behaviours or aspects of care that enhance or undermine the sense of dignity in patients with palliative care needs during illness experience and while receiving health services.
3. Critically assess the findings against the Dignity Model dominant in the western literature.

### Review method

An integrative method was adopted for this review [17]. An integrative review combines studies with diverse methodologies bringing forward a better understanding of complex phenomena. The inclusiveness of various research designs facilitates the building of comprehensive evidence that can be used to guide clinical practice and health policy. This openness to diverse methodologies allows the clear definition of concepts, appraisal of the existing evidence, and identification of knowledge gaps [17]. To enhance the rigor, a systematic and explicit methodology is applied throughout the review process, informed by the guideline set by Whittemore and Knafl [17] as follows:

1. Problem identification
2. Literature search
3. Data evaluation
4. Data analysis
5. Conclusion
6. Verification

### Literature search

The SPICE (Setting, Perspective, Intervention/ Phenomenon of Interest, Comparison, Evaluation) framework, that is appropriate for explorative questions, is applied to dissect the research question into elements, expand search terms, and specify study eligibility criteria for inclusion [18]. The SPICE elements of “perspective”, “comparison” and the “outcome/ evaluation” are not included in the search strategy to broaden the scope and optimize sensitivity [19]. Elements of the SPICE as employed in the research question are elaborated in Table 1 below.

**Table 1:** SPICE Framework

**Setting**	Palliative care in Middle Eastern countries
**Perspective**	Adult patients, health care providers, family caregivers, or any other member in the palliative care team.
**Intervention/ Phenomenon of Interest**	Studies that focus on the phenomenon of personal dignity.
**Comparison**	Having impaired dignity
**Evaluation /outcomes**	The perceived outcome of dignity or loss of dignity.

### Definition of key terms

- Dignity- “the quality or state of being worthy, honoured, or esteemed” [20].
- “Patient with palliative care needs”- is the individual suffering from a life-threatening condition that holds no possibilities of remission or cure. This encompasses patients suffering from a diverse incurable or progressive long-term illnesses such as cancer, organ failure, or degenerative diseases, who require physical, psychosocial, and spiritual care [21].
- Middle-Eastern countries sometimes termed as Eastern Mediterranean countries including the following 18 countries: Afghanistan, Bahrain, Cyprus, Egypt, Israel, Iran, Iraq, Jordan, Kuwait, Lebanon, Oman, Palestine, Qatar, Saudi Arabia, Syria, Turkey, United Arab Emirates, and Yemen [22].

### The search strategy

A preliminary scoping search was undertaken to be familiarised with the common terms associated with ‘dignity’. After consulting with a health librarian, a comprehensive search strategy was designed and applied to the selected four health databases; EMBASE, PsycINFO, PubMed, Cumulative Index to Nursing and Allied Health Literature (CINAHL) Complete, hosted by EBSCO platform. These databases are reported to retrieve a broad literature on palliative care [23] and are often chosen in other palliative care reviews [7]. Keywords, medical subject headings (MeSH) and multiple synonyms such as, “dignity”, “respect”, “self-concept”, “self-esteem”, “palliative care”, “terminally ill patients”, “life-threatening”, “end of life”, “dying”, “Arab”, “Muslim”, and “Mediterranean” were used simultaneously to search the titles and abstracts of captured papers. See Table 2.

**Table 2:** Search terms and strategy used in CINAHL database, keywords, and Mesh

Subject group	Search terms used
Dignity	TI ((Dignity OR dignified OR respect* OR person#hood OR “self-concept” OR “self-esteem” OR Distress* OR ((attitude OR good) N2 (death OR dying OR illness))) OR
	(MM “Human Dignity”) OR (MM “Respect”) OR (MM “Self-Concept”)
Palliative	TI (((palliat* OR terminal* OR hospice OR dying OR death) N2 (patient* OR experience* OR care OR phase OR prognosis OR ill* OR cancer)) OR “end of life” OR end-stage OR life threatening OR life limiting OR (final OR last) N2 (day*) OR “advanced cancer” OR
	(MM “Palliative Care”) OR (MM “Terminal Care”) OR (MM “Hospice Care”) OR (MM “Terminally Ill Patients”) OR (MM “Death”)
Middle East countries	TI ((Cypr* OR Afghanistan* OR Bahrain* OR Iran* OR Iraq* OR Israel* or gaza OR ghazza# OR “west bank” OR Palestin* OR Jordan* OR Leban* OR Liban OR Syria* OR Oman* OR Qatar* OR Kuwait* OR Saudi* OR “Saudi Arabia” or Turk* OR UAE or “united Arab emirates” OR Egypt* OR Yemen* OR Mediterranean OR Muslim* OR Islam* OR oriental OR Arab* OR middle#east OR (((cultur* or multicultur*) N2 (divers* OR chang*)))
	(MM “Culture”) OR (MM “Cultural Diversity”) OR (MM “Middle East”) OR (MM “Islam”) OR (MM “Arabs”)

The systematic search was conducted in December 2019 and the final search updated in December 2020. No date limits were set. The search strategy was modified on each database to adapt to its subject index or thesaurus terms [24]. The controlled vocabulary and free terms of each concept are combined in the search using the Boolean operator “OR”. Once each concept was combed, the searches were united with the Boolean operator “AND” to distil the final number of papers intersecting the three concepts. Supplemental methods of screening were employed to enhance the sensitivity of the search such as scanning the reference lists of review papers and included manuscripts, forward citation tracking, and hand searching of key journals [18]. The online journals, 1) Nursing Ethics, 2) The Medical Journal of Ethics & 3) Journal of Medical Ethics and History of Medicine, that publish relevant papers on dignity were screened for the last ten years. The retrieved manuscripts were vetted against the inclusion/exclusion criteria elucidated in Table 3.

**Table 3:** Summary table of inclusion and exclusion criteria

Framework	Inclusion	Exclusion	Rationale
Setting	A middle Eastern context The setting is that of palliative care inclusive of hospital, hospice, home-based, or community.	The countries of Algeria, The Comoros Islands, Djibouti, Mauritania, Morocco, Somalia, Sudan, Libya, Pakistan and Tunisia, though considered to be part of the eastern Mediterranean region, are excluded from the search.	They are geographically distant from the Middle- Eastern or Mediterranean area.
Perspective/ participants	Studies from the perspective of: a) Adult patients with life-threatening or advanced chronic illnesses such as cancer, or any organ failure (heart, kidney, liver pulmonary), and neurological disorders, who need palliative care attention. b) Health care providers like physicians, nurses, social workers, pharmacists, psychologists, dietitians, and chaplains c) Caregiver- or ‘carer’ described as an adult, aged 18 or over, who provides or intends to provide care for another adult needing care. It could be a family member, relative or other. This excludes people providing paid care or people providing care as voluntary work [25]. Studies limited to adult population – age > 18 years	Older adults or frailty or patients with dementia. Patients with mental health disorders. Paediatric population	Dignity in dementia or frailty entails addressing unique care needs especially in the advanced stages [26]. Many mental health patients suffer from marginalization and injustice implying a broader action on dignity than that of palliative patients [27]. Children within palliative care have unique dignity needs that differ from adults [28].
Intervention/ phenomenon of Interest	Studies that focus on dignity, the meaning or perceptions of dignity, dignity experiences, dignity related distress, loss of dignity, and dignified care. Studies related to barriers or enhancers of patient dignity will be included. Only empirical studies from peer-reviewed journals that follow quantitative, qualitative, or mixed-method design are included. Only English language papers were included.	Dignity discussed in relation to euthanasia, assisted suicide, assisted dying, right to die, death with dignity, or legislative aspects. Review articles, reports, editorials, commentaries, letters to the editor, books, dissertations, and papers that discuss dignity from legal or policy perspectives are excluded	Dignity is the key focus of the review Palliative care is understood as an approach that affirms life and does not hasten or postpone death. Its role is not only during the last days of life but from the time of diagnosis of an incurable disease. The goal is to improve the quality of life of those facing terminal illnesses as well as their family caregivers. For this reason, papers that tackle assisted suicide or euthanasia are outside the scope of the review and will be excluded [29]. Empirical studies are deemed appropriate to provide evidence on perceptions, influencing factors, or outcomes of dignity. Due to restricted resources, papers are limited to English language.
Evaluation/ Outcomes	The outcome of enhanced or impaired dignity as well as perceived benefits or threats will be examined. Studies that have dignity as a secondary outcome will also be included.	Outcomes other than dignity	

### Inclusion-exclusion criteria

#### Search outcomes & data extraction

**Fig. 1:**
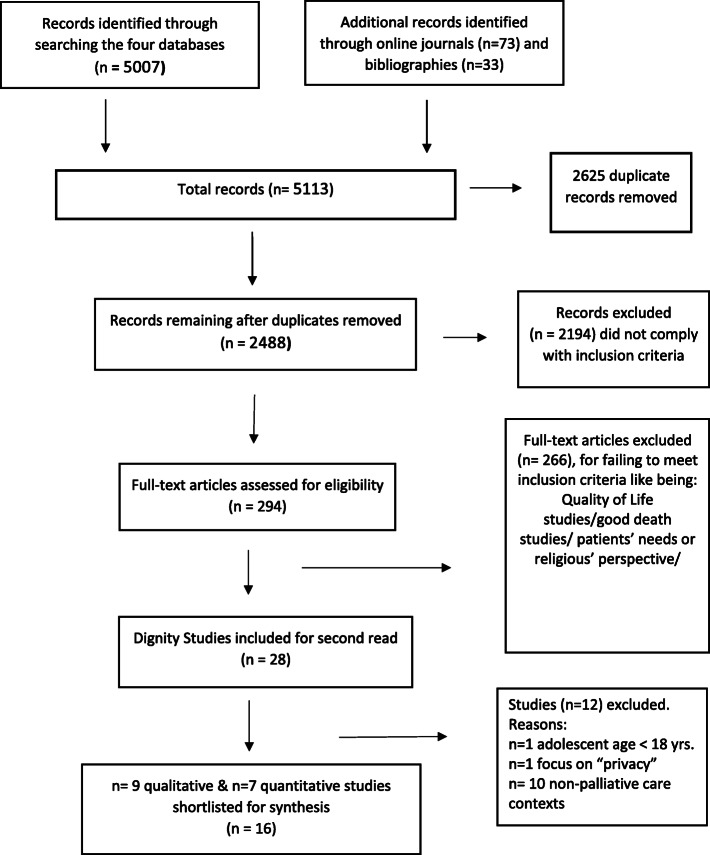
PRISMA Flow Diagram of the systematic review process

The search identified 5007 records from the four health databases that were transferred to an EndnoteX8 bibliography software for storing and removing duplicates. Seventy-three additional relevant papers were identified from the three journals and thirty-three from reference lists of review and included papers. After eliminating duplicates (*n* = 2625), 2488 papers were screened independently by two researchers (S.D.S.) and (R.S) by title and abstract against the inclusion criteria. The papers (*n* = 2194) that did not meet inclusion criteria were excluded. The potentially relevant papers (*n* = 294) were filed in Endnote for full manuscript review. These papers were categorised as those related to papers on patients’ needs, ‘good death’ papers, review papers, pure dignity papers, and so forth. A further 266 papers were excluded because many were not related to perceptions of dignity. Finally, the publications were narrowed to twenty-eight potentially relevant papers for a full script reading. In line with the Whittemore and Knafl [17] guidance, the data from the twenty-eight papers were reduced and displayed in an excel sheet in the form of a table. The table delineated the main characteristics of each paper such as the author, date of publication, country of origin, discipline, study design, aim, participants, context, sample size, data collection, data analysis, and findings. In the finding’s column, actual quotations from the primary papers were incorporated to preserve their wholeness. The tabular display allowed the visualization and the iterative studying of the papers to identify their unique characteristics. A second reviewer independently extracted the data from 50% of the included papers (R.S.) to validate the process and relevance of the records. After discussion and consensus with the wider team (NP, YS and RS), 16 dignity papers were included for final synthesis. The PRISMA chart in Fig. 1 displays the process.

### Data evaluation

The quality of the included sixteen papers were evaluated using the Hawker appraisal tool [30] that has broad criteria appropriate for appraising research from different paradigms and commonly used in palliative care reviews [31, 32]. Though the papers were evaluated and scored, no manuscript was excluded for its weak score. Instead, the impact of each paper was reported with a critical assessment in the synthesis [33].

### Data analysis

The data analysis stage involved categorizing, coding, and summarizing, of the included 16 papers to facilitate the organisation and reduction of the literature in a frame. The reports were primarily grouped according to their research design, and those that discussed perceptions of dignity, facilitators, barriers, or health outcomes of dignity. Codes were developed, such as “personal values of dignity”, “health care related issues”, “communication at end of life”, from each paper and arranged in a matrix to allow clear visualisation and comparison of patterns or variations. The codes were collated so that similar ones, such as the cleanliness of the hospital rooms, private space, quick service, and noise in the units, were assembled to generate a common theme, for instance, health facilities. Non-conforming codes were interrogated to unpick underlying social values like excessive treatment, distress in female patients, or retaining hope in the face of grave health condition. The analysis was an iterative process involving merging of codes and creating themes and subthemes to achieve a higher level of interpretation and abstraction (Table 4). Seven interrelated themes were developed that incorporated most of the codes. The conclusion was synthesized by integrating the themes and building a logical chain of how dignity is understood, enhanced or threatened that developed an original knowledge of dignity beyond surface description [17].

**Table 4:** Analytical framework of the developed themes on patient dignity

Themes	Subthemes	Papers
Maintaining Privacy & Secrecy	*Enablers* - Personal space/ rooms/ separation curtains - Decent hospital gowns - Knocking at the door before entering patient room - Private space to take care of daily bodily needs - Gender sensitive health care services - Concealment of medical condition and personal information - Secrecy of lifestyle or practices *Stressors* - *Nudity or exposure of body parts during physical* *examinations* *- Questioning about personal matters by nurses* *- Recurrent interaction with health care providers*	de Voogd et al., 2020 [34]; Bagherian et al., 2019 [35]; Bidabadi et al., 2019 [36]; Korhan et al., 2018 [37]; Bagheri et al., 2018a,b [38, 39]; Mehdipour-Rabori et al., 2015 [40]; Borhani et al. 2016 [41]; Sharifi et al., 2016 [42]; Borhani et al., 2015 [43]; Avestan et al., 2015 [44]; Bagheri et al., 2012 [45].
Gentle Communication	*Enablers* - Recognizing personal values - Individualized dialogue - Informing the patient about the treatment and required lifestyle changes - Kind & compassionate nursing care - Maintaining /respecting religious rituals during illness and hospitalization - Gentle disclosure of truth - Retaining a glimpse of hope in health-related dialogues. - Empathy: Being in the patient’s shoes - Personal view of self and life - Belief system and relation with God *Stressors* *- Bluntly disclosing the truth about the diagnosis* *- Excessive treatment of dying patients* *- Communication that implicates blaming, too* *much pity, and superior* versus *inferior* *relationship* *- Harassment & abuse*	de Voogd et al., 2020 [34] Bagherian et al., 2019 [35]; Bidabadi et al., 2019 [36]; Korhan et al., 2018 [37]; Sharifi et al., 2016 [42]; Borhani et al., 2016 [41]; Borhani et al., 2015 [43]; Hamooleh et al., 2013 [46]; Bagheri et al., 2012 [45].
Abundance of Resources	*Enablers* - Affording the needed medical resources - Availability of basic resources and facilities - Maintaining employability - Education, training, problem-solving skills, prior experience - Purposeful life, being worthwhile, maintaining social role - Charity aids - Maintaining a clean environment in the hospital, clean and private lavatories/ rooms, good lighting *Stressors* *- Shortage of health-sustaining needs like* *medications* - *Shortage of medical staff* *-Economic instability & uncertainty* *- Depending on family and friends* *- Young individuals are more vulnerable*	Bagherian et al., 2019 [35]; Bagheri et al., 2018a [39] ; Shahhosseini et al., 2017 [47]; Mehdipur et al., 2015 [40]; Avestan et al., 2015 [44]; Sharifi et al., 2016 [42]; Borhani et al., 2016 [41]; Borhani et al., 2016 [41]; Bagheri et al., 2012 [45].
Family Support	*Enablers* - Presence of family during hospitalization - Allowing visitations - Respect to family caregiver’s needs at the hospital - Family involvement in discharge planning, plan of care, and decision making - Adherence to treatment regimen, symptom relief and ability to seek medical help - Presence of social support when living with family and friends - Sense of medical, physical, and spiritual security - Maintaining social role - Community support to patient / empowering policies *Stressors* - *Living alone* *- Being cared for by professionals instead of* *family members*	de Voogd et al., 2020 [34]; Bagherian et al., 2019 [35]; Bagheri et al., 2018a [39]; Korhan et al., 2018 [37]; Shahhosseini et al., 2017 [48]; Amininasab et al., 2017 [49]; Mehdipur et al., 2015 [40]; Sharifi et al. 2016 [42]; Borhani et al., 2016 [41]; Borhani et al., 2015 [43]; Hamooleh et al., 2013 [46].
Physical Fitness	*Enablers* - Physical independence, being in control - Low burden & minimal medical complications symptom *-* Higher dependence on others deteriorates communication with friends and family *Stressors* *- Recurrent hospitalization* *- Uncertainty/ Insecurity* *- Burden on the family* - *Stigmatization* *- Losing social status* *- Minimizing the chances of getting married*	Bagheri et al., 2018a [39]; Bagheri et al., 2018b [38]; Korhan et al., 2018 [37]; Hosseini et al., 2017 [47]; Shahhoseini et al., 2017 [48]; Sharifi et al., 2016 [42]; Mehdipur et al., 2015 [40]; Avestan et al., 2015 [44]; Bagheri et al., 2012 [45].
Reliable Health Care	*Enablers* - Expert medical staff who provide error-free care - Prompt attention to patient needs - Comprehensive care that attends to the whole person - Kind nurses - Well staffed and managed ward - Health care providers who are neatly groomed and follow hygienic measures - Sustaining the physical body till the last days of life - Silence in intensive care units *Stressors* *- Reductionist practice* *- Lack of motivation from the health care providers* *-Pointless treatment*	Bagherian et al., 2019 [35]; Bidabadi et al., 2019 [36]; Korhan et al., 2018 [37]; Borhani et al., 2016 [41]; Borhani et al., 2015 [43]; Hamooleh et al., 2013 [46]; Bagheri et al., 2012 [45].
Social justice	*Enablers* - Equal care irrespective of social, economic, or medical status - Equal opportunities in life - Mutual respect and trust between patient and health care team - Mindful communication *Stressors* - *Discrimination* *- Injustice* *- Discrepancy between perceived values and the* *actions of health care providers’* - *Bureaucracy in the hospital governance/ strict* *regulations* *- Use of improper language* *- Paternalistic attitude*	Bidabadi et al., 2019 [36]; Korhan et al., 2018 [37]; Sharifi et al., 2016 [42]; Shahhosseini et al., 2017 [48]. Mehdipur et al., 2015 40; Borhani et al., 2015 [43]; Bagheri et al., 2012 [45]

### Findings

#### Characteristics of the studies

Sixteen papers that represented fourteen primary studies were included in the analysis. Fourteen of them came from the Republic of Iran, one from Turkey, and one from Netherlands that discussed how Turkish, Moroccan, or Surinamese patients understand important aspects of dignity; Fifteen studies were conducted by nurse researchers indicating the high significance of the concept of patient dignity in the nursing profession. Of the 16 dignity papers, 12 focused on patient perceptions. The remaining papers explored nurses, physicians, and hospital staff perceptions. One paper discussed dignity from the family caregivers’ perspective. The patients’ medical diagnoses ranged from heart disease (*n* = 7), to cancer (*n* = 4) to multiple sclerosis (*n* = 1) and mixed (n = 1). Studies on patients with other advanced chronic conditions that could also have palliative care needs (such as end-stage organ failure) was only one. Most of the studies (*n* = 14) were published in the past five years, and the rest from 2012 to 2013, showing that the concept of dignity is evolving in the Middle East and has gained attention more recently. The research designs were quantitative (n = 7), and qualitative (*n* = 9) in nature. Most of the qualitative papers employed a conventional content analysis method, one paper adopted phenomenology [37], one critical ethnography [36] and one thematic analysis identifying a thematic framework [34]. Whereas the quantitative studies were all descriptive, or descriptive-correlational in nature. See Table 5 for a summary of the characteristics of the 16 papers.

**Table 5:** Summary characteristics of the included papers (*n* = 16)

**Study participants**	Most studies (*n* = 12) focused on the perceptions of patients only; two papers studied nurses’ and physicians’ perceptions: Bidabadi et al. (2019) [36], Korhan et al. (2018) [37]; one on solely nurses’ perceptions: Hamooleh et al. (2013) [46]; and one on patients’ & relatives’: de Voogd et al. (2020) [34]. The paper by Borhani et al. (2016) [41] focused on patients and hospital staff.
**Country of Publication**	Fourteen studies were published in Iran only one from Turkey (Korhan et al. 2018) [37], and one from the Netherlands (de Voogd et al. 2020) [34].
**Date of Publication**	Most (n = 14) publications were during years 2015–2020; only two were before the year 2015 Bagheri et al. (2012) [45] and Hamooleh et al. (2013) [46].
**Setting**	
Cardiac unit	Bagheri et al. (2018a,b) [38, 39], Amininasab et al. (2017) [49], Bagheri et al. (2018a,b) [38, 39], Bagheri et al. (2012) [45]
Cardiac Surgery Intensive Care Unit	Bidabadi et al. (2017), Mehdipour-Rabori et al. (2015) [40], Borhani et al. (2016) [41], Borhani et al. (2015) [43]
Palliative care	Korhan et al. (2018) [37], Hosseini (2017) [47], Avestan et al. (2015) [44], Hamooleh et al. (2013) [46], de Voogd et al. (2020) [34].
Internal medicine	Bagherian et al. (2019) [35], Shahhoseini et al. (2017) [48]
Multiple sclerosis society	Sharifi et al. (2016) [42]

Each study contributed to the synthesis with several themes. The features of the included papers are organized in Table 6.

**Table 6:** Features of the included studies

Author, Date & Country of Publication	P**urpose**	R**esearch** D**esign**	P**articipants**/ C**ontext**	T**hemes**/ F**indings**
Studies from the patients’ perspective				
Bagherian et al., 2019 [35] Iran	To evaluate the concept of dignity from the perspective of Iranian cancer patients.	Semi-structured interviews using Qualitative Content Analysis.	Sixteen Hospitalized cancer patients > 18 years, (5 men & 11 women)	• The key elements of dignified care were the preservation of personal space and privacy, respect for values, and the provision of adequate moral support to patients.
Bagheri et al., 2018a,b [38, 39] Iran	To determine the relationship between illness-related worries and social dignity of patients with heart failure.	Descriptive- analytic. Two questionnaires used: illness-related Worries Questionnaire (IRWQ) and Social Dignity Questionnaire (SDQ)	Total of 130 heart failure inpatients from cardiac hospital wards.	• A significant correlation was observed between illness-related worries and social dignity. So that, decrease in physical, mental, cognitive worries and worry about future of disease improves communication and decreases the sense of burden to other and vice versa
Bagheri et al., 2012 [45] Iran	To investigate perceptions of patient dignity and related factors in patients with heart failure.	Qualitative semi-structured interviews using qualitative content analysis method described by *Hsieh and Shannon*.	Twenty-two heart failure inpatients in cardiac hospital wards	Dignity means: • being considered as a *unique human being, being treated with respect, and having forgiveness*. • Factors enhancing or threatening patient dignity were classified into two main categories: ‘patient/care index’ and ‘resources’. • Intrapersonal features (and interpersonal interactions) were classified as components of the patient/care index category. Human resources were classified as components of the *resource’s category*
Bagheri et al. 2018a,b [38, 39] Iran	To investigate factors related to dignity in patients with heart failure and to test the validity of the Dignity Model.	The study had a descriptive-correlational design. Using four questionnaires.	Hundred and thirty hospitalized heart failure patients.	• The research model is fit in patients with heart failure, and dignity related factors are in correlation with each other. • Social dignity is the biggest factor in the dignity of patients with heart failure. ‘Dignity conserving repertoire’ and ‘Illness related worries’ (affected by the frequency of hospitalization and age) also affect dignity.
Mehdipour-Rabori et al., 2015 [40] Iran	To investigate the status of human dignity in patients with cardiovascular disease (CVD)	Cross-sectional descriptive design. Two questionnaires used to collect data: A demographic questionnaire, and the Patient Dignity Inventory (PDI).	Two hundred cardiac patients hospitalized in Coronary Intensive care units	• Significant relationship between *gender and emotional problems related to human dignity;* women feel more problems associated with human dignity than men. • Significant relationship between the number of *hospitalizations and problems related to patient dignity*. • Significant correlation between *living alone and problems associated* with human dignity
Amininasab et al., 2017 [49] Iran	To determine the relationship between human dignity and medication adherence in patients with heart failure.	Cross-sectional descriptive design. Data were collected using demographic and clinical questionnaires, PDI, and the Morisky Medication Adherence Scale (MMAS-8).	Three hundred hospitalized patients with heart failure.	• A negative relationship exists between medication adherence and a threat to human dignity (correlation coefficient r = − 0.6, significance level *P* < 0.001). The higher the score of threat to dignity, the lower the medication adherence.
Shahhoseini et al. 2017 [48] Iran	To determine the sources of dignity-related distress from the perspective of women with breast cancer undergoing chemotherapy.	Cross-sectional study design. Data collected using demographics and the PDI.	Two hundred seven patients with breast cancer undergoing chemotherapy.	• Patients mostly concerned about the distress caused by disease symptoms, existential distress, peace of mind, dependency, and social support. • The patients undergoing mastectomy expressed higher level of social support and dependency distress than patients not undergoing the surgery. • Income satisfaction had a significant relationship with Existential Distress and Symptom Distress.
Borhani et al. 2016 [41] Iran	To investigate facilitators and the factors threatening the dignity of the patients with heart disease.	Qualitative semi-structured interview. Content analysis constant comparative method with inductive approach used for analysis.	Twenty hospitalized cardiac patients from the cardiac intensive care units and 5 personnel.	• Care context is important for patients’ dignity and includes human and physical environments; also, • Safe holistic care (Meeting the needs of patients in the hospital and after discharge; Creating a sense of security) are important aspects affecting the dignity of patients. • Dignity is impaired when the staff do not perform effective communication like Respectful Relationship, and Involvement of the Family in the Health Team.
Sharifi et al. 2016 [42] Iran	The study aimed to investigate factors affecting dignity of patients with MS in the society.	Qualitative semi-structured interviews; using conventional inductive content analysis.	Thirteen patients with multiple sclerosis.	Factors affecting patient’s dignity classified into personal and social factors. • Personal factors include the four subcategories of *patient’s communication with self, patients’ knowledge, patient’s values and beliefs, and patient’s resources*. • Social factors also include four subcategories of others’ communication with patients, social knowledge, social values and beliefs, and social resources.
Borhani et al. 2015 [43] Iran	To explore the meaning of patient dignity.	Qualitative- interviews using content analysis	Sixteen hospitalized heart patients admitted to the cardiac intensive care units.	Two main categories; Basic dignity and Transcendent dignity. • Basic dignity is related to physical and psychological health. It included subthemes of human security, comprehensive care, education and awareness, respect, effective communication, and privacy. • Transcendent dignity aims to create a full human with spiritual health. Subthemes such as trust, gratitude, appreciation, and spiritual growth were included in this category. • Findings showed that some of the participants were not satisfied with the basic dignity alone, and they were seeking transcendent dignity.
Avestan et al. 2015 [44] Iran	To explore cancer patient perceptions of respecting their dignity and related variables.	Descriptive Correlational design. Data collected through demographics and then the Dignity Inventory (PDI).	Two hundred and fifty cancer patients.	• Perceived dignity violation in illness-related concerns. • The sense of anxiety and depression, uncertainty regarding the disease and treatments, and worrying about the future were the main symptoms of lack of preserved dignity in this sub-scale.
Hosseini et al. 2017 [47] Iran	To assess the association between the status of patient dignity and quality of life (QOL) in terminally ill patients with cancer.	Descriptive correlational study. Data collected using the (PDI) and the Persian version of the (EORTC QLQ-C30)	Two hundred and ten end-stage cancer patients (102 men and 108 women).	• High dignity status in terminally ill patients associated with higher QOL in terms of functional intactness and lower symptom distress.
Studies from the health care providers’ perspective				
Bidabadi et al., 2019 [36]. Iran	To uncover the cultural factors of power that impeded maintaining patients’dignity in the cardiac surgery intensive care unit	Critical Ethnography- Observations; data analysed conducted hermeneutically and reconstructively.	Nurses, physicians, internal medicine specialists, cardiac surgeons, anaesthesiologists, auxiliary nurses from an adult cardiac surgery unit	• Factors that impeded maintaining patient dignity were Reductionism, Instrumental objectified attitudes*.* • A value - Action gap existed in adhering to the human equality principle. This theme consisted of two subthemes: ‘authoritative behaviours’ and ‘Blaming the patients.
Hamooleh et al., 2013 [46]. Iran	To explain nurses’ perception about ethics-based palliative care in cancer patients.	In-depth interviews using Qualitative Content analysis	Nurses taking care of cancer patients.	Ethical palliative care from the nurse’s perspective had three themes: • ‘human dignity’, ‘professional truthfulness’ and ‘altruism’. • Human dignity had 3 sub-categories consisting of ‘respecting patients’, ‘paying attention to patient values’ and ‘empathizing’.
Korhan et al., 2018 [37] Turkey	To determine the approach to human dignity that nurses and physicians have while providing palliative care	Phenomenology - Semi-structured interviews using a guide prepared by the investigators. Data analysis was guided by the *Colaizzi method.*	Physicians & Nurses in the Palliative Care Department of Training & Research Hospital	Eight Themes and 43 subthemes: *Decision for patients to know their diagnosis; Ensuring the quality of end-of-life care; Care procedures carried out on patients; Adequate provision of medical care services; Prioritization in palliative care; Pointless treatment in palliative care; Views on the concept of respectful care; Views on palliative care.* The results showed that there was a lack of awareness of ethical, medical, and social responsibilities that led to violation of human dignity.
Studies from patients’ and Caregivers’ Perspective				
de Voogd et al. 2020 [34]. Netherlands	1) To gain insight into what patients and their relatives with a Turkish, Moroccan, or Surinamese background, find important to preserve their dignity in their last phase of life and 2) how care professionals can preserve and strengthen sthe dignity of these patients.	Qualitative thematic analysis	Twenty-three patients with a Turkish, Moroccan or Surinamese background and 21 relatives.	• Dignity encompassed surrender to God’s will and meaningful relationships with others, rather than preserving autonomy. • Surrender to God meant accepting the illness and performing religious practice. • A meaningful relationship meant being assisted or cared for by family members and maintaining a social role. • Professionals could preserve dignity by showing respect and attention; guaranteeing physical integrity, hygiene, and self-direction; and indirect communication about diagnoses and prognoses.

#### Themes

The seven themes, as generated from the analysis, related to the understanding of patient dignity in illness and while receiving care is described here.

- Maintaining Privacy & Secrecy: Paying attention to patients’ privacy during care and being considerate of keeping personal information confidential was seen as respecting patients’ sense of dignity. Patients and health care providers agree that having a personal space like a private room in the hospital with separation curtains, personal toilet, a decent hospital gown that does not expose body parts, is considered essential to preserving patient dignity [34, 35, 37, 43, 45]. Crowded spaces and proximity to other patients that prevent performing basic self-care activities comfortably such as using the bedpan or washing diminish dignity. Entering patient rooms without prior knocking, particularly when female patients did not have their head veils in place [43], or loudly announcing frequency of defecation, offended patient privacy [36]. Unannounced nursing or physician ward rounds were regarded as disrespectful and gave way to indecent body exposure to patients not only in a palliative context but also in any clinical setting irrespective of specialty [50, 51]. Questions from the nursing staff that probed into the patient’s personal life such as how the illness had impacted intimacy was regarded to be unethical, rude, and infringing on patient’s privacy [35]. Recurrent interactions with health care providers and hospitalisations increased patient vulnerability and the risk of losing personal and physical privacy that threatened dignity [38, 40, 44]. This could be related to the imperfect health care system and increased vulnerability of the patients.
  Maintaining confidentiality of the medical diagnosis and limiting disclosure to a restricted family member is regarded of the utmost importance to patients [41, 43], to the extent that some individuals would deliberately search hospitals that are far from their residencies to maintain discretion [35]. Informing others about their illness carried the risk of stigmatisation or social isolation that risked losing societal status. This was true for patients with chronic non-cancer and cancer conditions. For instance, revealing the diagnosis of cancer or multiple sclerosis placed the patient at risk of being judged or looked down upon by the community, and missing out chances of getting married for both men and women [35, 42] which were key elements to maintain dignity. Secrecy regarding lifestyle or certain practices, such as the use of opium in male patients, was crucial to maintain a sense personal dignity [43].
- Gentle Communication: too much pity, blaming, or bluntly disclosing bad news is regarded disrespectful and damaging to personal pride and dignity. Paying attention to patient values, empathising, and delivering what he/ she is ready to hear is considered compassionate and enhancing of dignity [34, 35, 41, 43, 45, 46]. Expressions of pity are regarded to be patronising to the patient by affirming the patient’s weak position. Patients valued communicating hope and considered it superior to truth-telling, even in conditions of poor prognosis [34, 37]. Blunt disclosure of diagnosis is undesirable by the patients and health care providers with the belief that maintaining a glimpse of hope promotes patient dignity [35, 37]. Abstaining from talking about the seriousness of the condition and believing in the person’s predestined fate, surrendering to God’s will, is a coping mechanism to maintain dignity [34]. Despite the nondisclosure of the diagnosis, nurses kept informing patients about the inevitable adverse effects of cancer medications like hair loss [46] indicating that patients often were inherently aware of their condition, upholding a glimpse of truthfulness. Amid hospitalization and illness, patients were keen on following their religious rituals such as prayers to preserve their identity and dignity [34]. For instance, a patient was upset to have missed morning prayer due to receiving a sleeping pill the night before [41, 43]. On the organisational level, bureaucracy, strict regulations [52], and communication that is not driven by patient needs, but is rather paternalistic or routine-oriented, reduces patients’ experiences of being valued [36]. For instance, strict visiting hours, excluding family members, banning the use of telephones, restricting patient freedom undermine the sense of worth and trust among patient and health care provider, consequently diminishing overall satisfaction with care and sense of dignity [41, 43, 45]. Attentive communication that is engaging, and mindful of individual preferences assures a dignified experience [43]. Though communication implies that dignity is a dynamic relational process mediated by social interactions and environmental factors, it is also an individual perception affected by how one views self-worth and communicates internally with self. For instance, self-blame, self-doubt and the belief that the illness is the result of personal deficiencies is harmful; whereas focusing on personal strengths and opportunities promotes dignity [42]. Perceptions of self-value are influenced by the individual’s understanding of their purpose in life, relationships to others, religious beliefs, and cultural norms. Faith is either an impetus for expression of resilience or a predisposition to succumb under the pressure of ill health and see its limitations as an unjustified loss that is to be borne [42].
- Abundance of resources: Economic burdens and overshadowing fears of not getting hold of the essential medical resources and health services due to unavailability or inaccessibility are damaging to personal identity and dignity [35, 38, 44, 48]. Recurrent admissions to the hospital are associated with high expenses, financial burdens, increased reliance on family support, and heightened patient vulnerability [38, 40, 44]. In contrast, economic prosperity is related to perceptions of being worthwhile, dignified, having a purposeful life, and a meaningful social role [48]. Patients with or without palliative needs valued the availability of sufficient medical resources and spacious facilities in the health care system, such as clean and separate lavatories, good lighting, and silence in the intensive care units [41, 45, 50]. Unequal or disparate care is practiced in times of shortage of resources, medical staff, especially nurses, who become overworked and unable to meet the entire patients’ needs [41]. Multiple sclerosis patients who could earn an income despite their disabilities felt more dignified and useful compared to those who were forced to resign [42]. In this regard, education and competencies, such as proficiency of languages, problem-solving skills, were protective to patients’ dignity because, amidst physical disability, it secured employment [42]. The inability to sustain economic steadiness is particularly frustrating to the young patients because idleness reaffirmed a sense of unfitness closing down on chances of achieving life goals, hence, shattering personal dignity through the inability to provide for oneself [42, 44].
- Family Support: family caregivers are regarded as a central block of support system to the patient, particularly during hospitalisation for women [38, 41, 48]. The patient anticipates family caregivers to be present, and be included in care planning, and discharge preparations [35, 41, 43]. Patients who were living alone or lacked a support system from friends and family had a higher sense of disease burden than those who were married or who enjoyed family support [40, 42]. The family offered a social security net, particularly for women, for whom maintaining a social role for instance of a “mother who cooks for the family” was an affirmation to self-esteem and personal dignity [34]. The role of the family caregiver extends beyond the acute hospital setting to offering care and assistance after discharge [34]. The family members helped patients adhere to treatment and lifestyle regimens, find symptom relief, and seek medical help when needed [41, 49]. For this reason, effective communication between health care providers and patient/family, that entails conveying explanations about the disease and its management, is regarded as crucial to empower the ill individual in making informed decisions and achieving improved outcomes thereby maintaining dignity [37, 41–43, 46]. Patients with chronic conditions desire the social environment to be responsive, engaged and understanding to their social needs to uphold independence, security, and wellbeing. A supportive community that empowers patients by offering accessible self-care facilities [43], comfortable housing, safe transportation means, economic stability, is regarded as dignified standards of living and much desired [41, 42].
- Physical Fitness: It is worth noting that functionality and low symptom burden foster patient dignity within the family context [38, 39, 47]. The capability to independently perform daily self-care practices [44], being physically in control of the body, are seen to promote self-concept acting as a protective agent to dignity [39, 40, 42, 45, 47, 48]. A correlational study by Hosseini, Rezaei [47] reported that higher physical functionality was associated with lower anxiety, enhanced sense of worth, better symptom control, autonomy, and overall better quality of life in end-stage cancer patients. Whereas reduced physicality diminished privacy, and changes in appearance brought forward feelings of loss of dignity and being a burden on friends and family [38–40]. Distressing physical symptoms and uncertainty of the health condition seem to be detrimental to mental wellbeing too, leading to excessive worrying, anxiety, and depression due to the inability to cope with the heightened sense of loss of control [40, 44, 47, 48]. Thus, focus on psychological and physical symptom management is highly perceived to maintain dignity in patients with palliative needs [37].
- Reliable Health Care: Expert medical staff with specialized knowledge and prompt attention to patients’ needs are regarded as essential to dignified health care irrespective of the clinical setting or patient diagnosis [35, 41, 43, 53]. Competent hospital staff members who are diligent, kind and attend to patient needs, despite the unfavourable conditions of the health care institution (high workload or shortage of staffing and resources) are appreciated by all patients [41, 43, 45, 51, 53]. Time given to assess and talk to patients and not only medicate is vital in the understanding of dignified care [37, 41, 46]. Whereas a reductionist order that objectifies human encounters to medical tasks and body organs instead of a holistic approach fails to protect patient uniqueness and personal dignity [36]. Safe and error-free services transmit a sense of security and assurance that no harm is incurred to the patient’s wellbeing [41, 43]. A sufficiently staffed ward that is run by health care providers who appear clean with proper simple attire, was seen to provide an effective and respectful communication that in turn, promotes patient dignity [41, 45]. Gender-sensitive care in the hospital is essential to female patients [35] as well as preferred in male patients to enable a dignified self-concept particularly when it is related to personal care [43]. While for patients who were at end of life, maintaining their physical needs till the last minute of life such as continuing artificial feeding, body hygiene, and pain management, were regarded highly essential in maintaining the dignity of the dying patient by nurses and patients with heart disease [37, 41, 46]. Though blindly following cure-oriented care was sometimes seen as controversial by health care providers and caused personal dilemma [37].
- Social justice: Patients expect to have just and equal care regardless of low social or economic rank. Being left behind, stigmatized or marginalized due to hardship, foreign nationality, or a medical condition such as addiction or contagious infection, does not foster personal dignity [45]. Though health care providers hold a firm conviction and oath of providing equal health care to all [37], there existed a gap between the values and actions of health care providers’ where services delivered are affected by consumers’ status [36]. A sense of injustice was reported in Sharifi, Borhani [42] when patients with multiple sclerosis, hoped and aspired to have equal opportunities for work, family life, marriage, and non-discriminatory mindset in society that could maintain personal identity and dignity. Humility, mutual trust, compassion, and the gratitude that springs from this altruistic human relationship are sought by patients that is perceived as transcendental dignity [43, 53].
  Being a female patient with palliative needs increases the emotional distress and instances of decreased dignity [40]. Wives with physical disabilities or weaknesses felt their husbands did not perceive them as complete partners due to their ailment. Some women suffered from disrespect, marital discordance, and had lost intimacy in their relationship [42]. Women often lost their peace of mind for maintaining their role as a wife or a mother in the family household [48]. Men, on the other hand, felt useless when they lost their jobs or were rejected for employment due to their health condition [42].

## Discussion

The purpose of this review was to integrate and critically synthesise the knowledge on dignity of patients with palliative care needs and how it is influenced during illness and as a health care consumer in the middle east. Dignity is threatened in illness, in both genders, maybe sometimes more in females, when individuals lose the ability to assert their traditional roles in the family or community [35, 42, 43]. Insufficient symptom relief, loss of functionality, and the uncertainty of outcomes [44, 47, 48] aggravate the sense of loss of social role. A patient’s personhood thrives within the protected boundaries of privacy, confidentiality, and preservation of sociocultural formalities that nurture a sense of security [35, 37, 41, 43, 45].

When consuming health services, personal dignity is fostered by the empathetic and compassionate interaction between the patient and the health care provider. The patient and family expect information about self-care practices delivered in a sensitive way respecting individual values and cultural preferences. Objectifying attitudes, non-engaging, or non-symmetrical communication between patients and health providers are perceived as disrespectful and a major opponent to dignified health care [36].

**Fig. 2:**
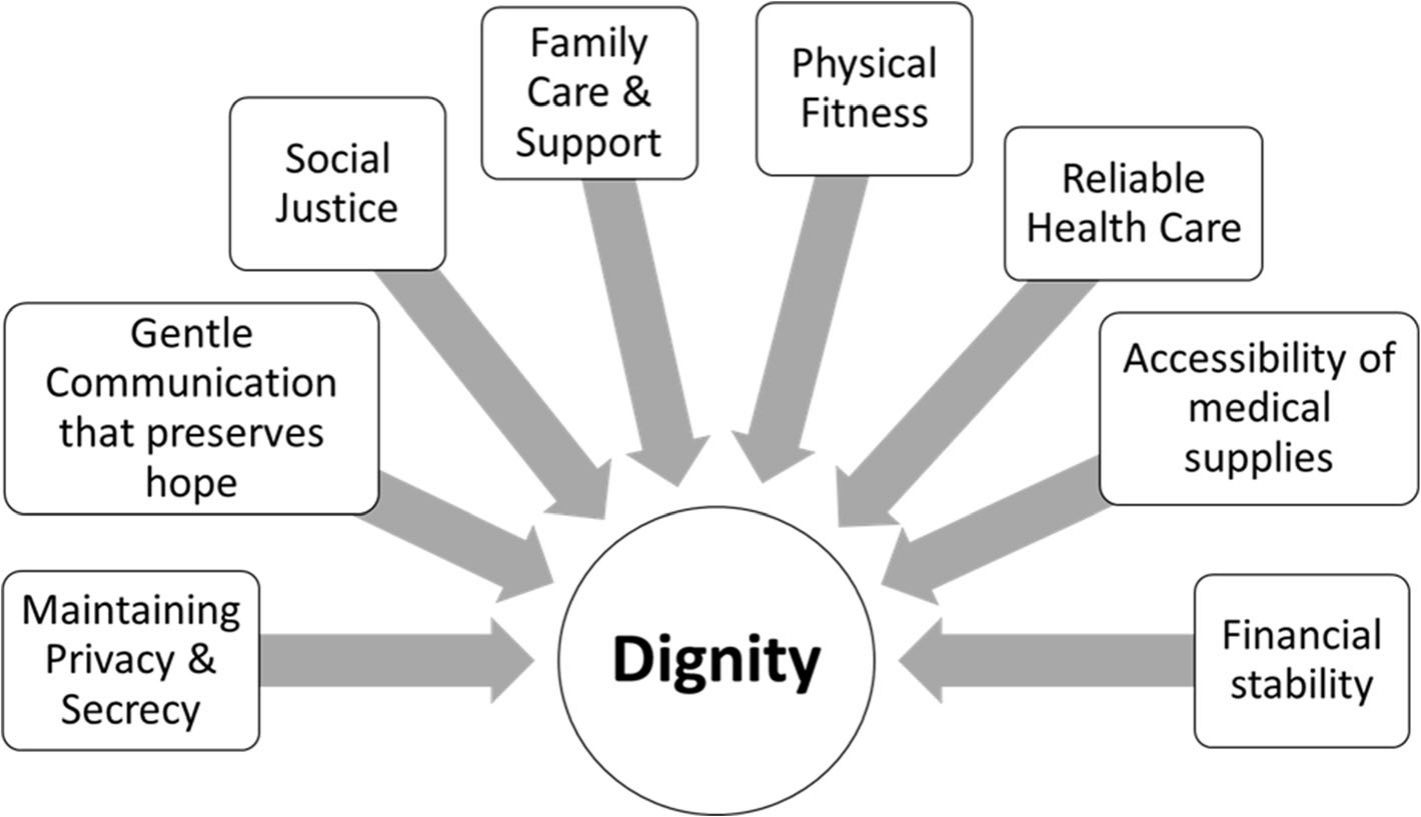
A preliminary model of dignity

Comparing these findings against the Chochinov’s empirical Model of Dignity [4], shows overall compatibility to its three domains in that the phenomenon of dignity is affected by illness concerns, shaped by personal outlook and resilience, as well as mediated by social relationships. Loss of physical or cognitive fitness appears to disturb patient dignity irrespective of geographical or cultural setting. Preserving social role and support seeking spiritual comfort and the capacity for independent body care maintained the pride and dignity of patients. The more the invasion of personal body space by caregivers and the open “uncivilized” discussion of body products such as the number of bowel movements, the more the disruption of privacy and thus personal dignity. Moreover, new themes like economic stability, availability of resources, reliance on religious faith, upholding hope for improved health, and the central partnering role of family members in the care of the patient emerged as distinct nuances in the understanding and preservation of patients with serious illnesses. Patient-centered palliative care and communication that pays attention to the values and spiritual beliefs of the patient ensures the creation of an empathetic space supporting the patient’s sense of worthiness [54]. In this aspect cautious disclosure of the diagnosis and prognosis to patients with terminal illnesses though may seem to be a conspiracy of silence, would respond to some patients’ or their families’ preferences of not discussing death or the end of life [55]. These themes reflect underpinning socioeconomic aspects and cultural values that shape the perception of dignity in illness in middle eastern culture. Self-determination and an individual’s need to control life and death events that is congruent to the dignity attributes in most western societies and northern America [6], is overshadowed with the collective decision making and the prayerful commitment to surrender to God’s sovereignty in controlling the illness and life destiny. In sickness, hope for a divine miracle of recovery or any improvement in health takes precedence to medical management. Gentle disclosure of the prognosis that entails an effective and skilful communication that is timely and tailored to patient wishes maintains aspiration for better health and is viewed as a source of moral support in futile conditions [35, 37, 46]. Telling the truth about the diagnosis and prognosis, though considered the reasonable practice in most westernized cultures to promote patient wishes [56], can be considered inapropriate in the eastern Mediterranean region. The unique themes emerging from this review are displayed in a preliminary model of Dignity in Fig. 2.

It is worth commenting that the phenomenon of dignity is not equally explored in all countries of the region and it represents predominantly an Iranian lens that has a majority Muslim-Shia population [57]. The Iranian health care system has been under significant funding constraints due to the strict economic sanctions [58]. This implies that poor political and economic conditions and scarcity of medical supplies deprive patients from attaining full health, leaving them feeling worthless and undignified [35, 36, 42, 59]. Thus, the findings cannot be extrapolated to the whole region of the Middle East but could be valid in countries where the health care sector is strained due to economic crisis, such as Lebanon, leading to disparities and unequal distribution of health care [60]. Also, it is worth noting that some of the collated studies came from similar research groups who have likely enriched their succeeding findings using similar participants and context limiting the generalizability of the review.

Most of the included qualitative studies in this synthesis adopted qualitative content analysis as a tool for data analysis. Content analysis mostly finds meaning through the recurring codes with the application of various levels of interpretations of manifest or latent concepts. It was unclear whether content analysis adopted in the research papers, is applied as a methodology or as a tool in the studies. Hence, the underpinning philosophical assumptions of the researcher, when explicitly stated in the manuscript, would reflect a better understanding of data analysis and findings [61, 62].

### Strengths and limitations

The strength of integrative reviews is that it allows a comprehensive grasp of a complex phenomenon by combining both quantitative and qualitative research studies. However, analysing the data and collating themes from diverse sources can be challenging [17]. All the pertinent studies were included regardless of their quality. Due to restraints in resources, only English language papers were included, excluding evidence reported in other languages. Most of the quantitative evidence is descriptive or correlational reporting the level of perceived dignity and identifying various factors, social and illness related, that affect personal dignity. On the other hand, qualitative papers uncover the personal understandings of the dignity phenomenon without the application of predetermined tools. The synthesis clarifies the meaning of dignity in patients with palliative needs by identifying common themes and tapping on subtle differences that are relevant to culturally competent health care practice and policy.

## Conclusion

This is the first systematic review that explores the multifaceted concept of dignity in patients with palliative needs in the Middle East. It presents a broad understanding of a sense of dignity that allows reflections on how personal, social, cultural, and economic forces influence sense of dignity and clinical practice. The studies included herein mostly explore patients’ or health care providers’ perspectives on dignity or factors augmenting or diminishing it. There remains a significant gap in the literature in the understanding of dignity from patients, health care providers’ and particularly family caregivers’ perspectives in the broader Middle East area beyond Iran. This review reveals that cultural awareness like the dominant role of family in patients with palliative needs, culturally sensitive skills such as indirect communications about the end of life, and accessibility of reliable services are essential aspects of personal dignity in some parts of the middle east.

## Data Availability

All data are available for the public in this manuscript.

## Abbreviations

EMBASE: Excerpta Medica dataBASE
Psych-Info: Psychological Information Database
PubMed: primarily processed the MEDLINE database (Medical Literature Analysis and Retrieval System Online)
CINAHL: Cumulative Index of Nursing and Allied Health Literature
SPICE: Setting, Perspective, Intervention/ Phenomenon of Interest, Comparison, Evaluation
PRISMA: Preferred Reporting Items for Systematic Reviews and Meta-Analyses
